# Study on Acoustic Emission Characteristics and Damage Mechanism of Wind Turbine Blade Main Spar with Different Defects

**DOI:** 10.3390/polym16233261

**Published:** 2024-11-23

**Authors:** Yanan Zhang, Shaojie Xue, Chuanyong Chen, Tianchang Ma, Bo Zhou

**Affiliations:** 1 School of Mechanical Engineering, Shenyang Ligong University, Shenyang 110159, China; 2 School of Energy Engineering, Zhejiang University, Hangzhou 310058, China; chen_cy@zju.edu.cn; 3 School of Mechanical Engineering, Shenyang University of Technology, Shenyang 110870, China; ma.tc@sut.edu.cn (T.M.);

**Keywords:** wind turbine blade, main spar, glass/epoxy composites, acoustic emission, damage mechanism, damage modes

## Abstract

This paper aimed to understand the AE signal characteristics and damage mechanism of wind turbine blade main spar materials with different defects during the damage evolution process. According to the typical delamination and wrinkle defects in wind turbine blades, the GFRP composite with defects is artificially prefabricated. Through acoustic emission experiments, the mechanical properties and acoustic emission characteristic trends of wind turbine blade main spar composites with different defects under tensile loading conditions were analyzed, and the damage evolution mechanism of different defects was explained according to the microscopic results. The results show that the existence of artificial defects will not only affect the mechanical properties of composite materials but also affect the damage evolution process of the materials. The size and location of delamination defects and the different aspect ratio of the wrinkle defects have a certain influence on the damage mechanism of the material. K-means cluster analysis of AE parameters identified the damage models of GFRP composites. The types of damage modes of delamination defects and wrinkle defects are the same, and the range of characteristic frequency is roughly the same. This study has important reference significance for structural damage monitoring and damage evolution research of wind turbine blade composites.

## 1. Introduction

**Figure 1:**
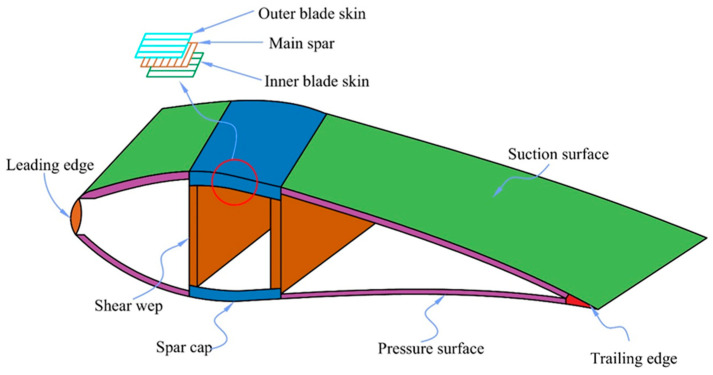
Profile structure of wind turbine blade.

The most important part of producing wind energy is the wind turbine blade, whose production costs make up roughly 23% of the unit cost [1]. The majority of wind farms are situated in isolated locations, making maintenance and monitoring of the turbines challenging. Inaccurate assessments made too late could result in catastrophic damage to wind turbine blades under demanding operating conditions, catastrophic accidents, and enormous financial losses. The profile structure of large wind turbine blades is depicted in Figure 1, and more than 90% of the components of wind turbine blades are composed of composite materials [2].

About 80% of the weight of the wind turbine blades is supported by the main spar, with the remaining 20% being supported by the leading and trailing edges [3]. The primary spar’s structure is laminate, and to increase its strength and stiffness, a high unidirectional strength glass fiber fabric is utilized as the reinforcing phase [4]. About 90% of the total brandishing stiffness is contributed by the main spar, and web plates provide internal support for the two shells to guarantee adequate structural stability [5]. In blade fracture accidents, the main spar fracture is the most common fracture form. The main spar’s local stress concentration can be caused by the combination of inadequate vacuum perfusion and the main spar’s lay-up properties, which can readily result in blade manufacturing errors. Defects will decrease the material’s fatigue resistance, which will exacerbate damage initiation and expansion under complex random loads and hasten the main spar’s fracture process. Currently, blade quality is mostly in danger due to manufacturing faults in primary spars, which also play a significant role in early failures and blade fracture accidents [6]. Delamination, wrinkles, air bubbles, and a lack of adhesive are examples of defects that can occur during the main spar laminate preparation process because of the reinforcement, matrix, and interface between them [7]. For example, the fiber cloth is not set flat during the cloth layer laying process, which leads to the formation of wrinkle faults. Because the fiber and resin have different thermal expansion coefficients and rates of moisture absorption, there is a chance that they will expand to different degrees during the curing process, which will ultimately result in the formation of lamination defects. In addition, poor air inflow or exhaust during vacuum perfusion leads to poor resin infiltration of the molded leaves [8].

**Figure 2:**
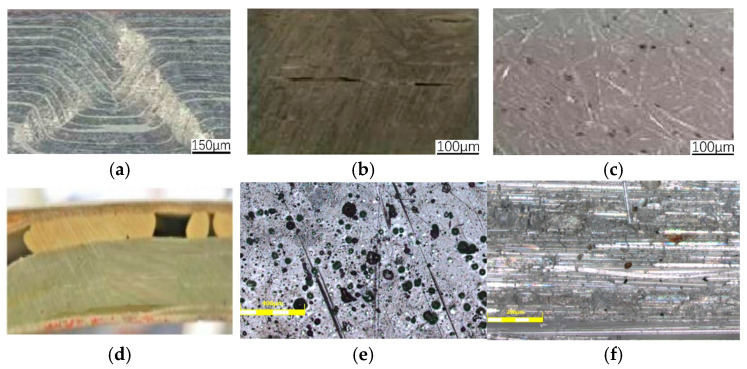
Typical manufacturing process defects of wind turbine blade main spar structure: (**a**) wrinkle defects; (**b**) delamination defects; (**c**) air bubble defects; (**d**) lack glue defects; (**e**) pore defects; (**f**) inclusion defects [16].

The combined influence of unstable components like delamination is the primary cause of blade damage. The sort of blade defect that most affects the structural performance of the blade is the delamination defect. Lamination defects are among the most prevalent types of defects in blades because the composite material used to make blades is made up of multiple layers, making it simple for defects to occur between them [9]. Wrinkle defects are a common main process defect found in many manufacturers’ goods because they are reasonably easy to arise in the blade layering process and can have a significant impact on the reliability of composite material products [10]. In particular, if there are wrinkle defects in the root, trailing edge, main spar, and other structural areas, in the operation of wind turbines, the interlayer cracking of fiber-reinforced composite materials in the wrinkle area will cause lamination damage of blades in serious cases [11]. At present, according to the characteristics of quality accidents in blades, wind power blades basically have three major risk sources: raw materials, manufacturing process defects, and design factors. Regardless of the maintenance faults or scrapping caused by quality reasons, process defects occupy a dominant position [12,13]. Typical manufacturing process defects of the wind turbine blade main spar structure are shown in Figure 2.

We aim to investigate the influence of manufacturing defects on the healthy life of wind power blades. At present, due to the limitations of manufacturing levels and process technology, the manufacturing defects of wind power blades cannot be completely avoided. Therefore, it is very important for the structural health monitoring of wind power blades to analyze the signal characteristics, damage modes, and damage mechanisms of different defects with the help of sensors. Over the recent years, a remarkable number of scientific papers demonstrate the capability of AE in nondestructive testing (NDT), structure health monitoring (SHM), condition monitoring (CM), and fault diagnosis for RE generation, transmission, transformation, and storage systems [14]. Due to the high sensitivity of acoustic emission detection, it can effectively identify and monitor the weak matrix cracks and fiber damage components and characteristics in structures. The generation of acoustic emission signals changes with the stress, time, and temperature of the tested component, so dynamic information of structural damage and damage evolution with the above variables can be obtained, and long-term continuous state monitoring can be conducted. At present, acoustic emission monitoring technology has been widely used in damage monitoring, damage source location, and damage identification [15].

In the study of damage evolution, Nikhakht et al. [17] conducted a series of experiments on glass fiber reinforced plastic material samples with different layerings through acoustic emission data analysis and microscopic imaging methods. The results show that matrix cracking is the first failure mode, and its frequency ranges from 50 kHz to 200 kHz. The damage evolution depends on the fiber orientation. In addition, the load displacement curve, acoustic emission data, and microscopic imaging results have a good correlation in the initial, evolution, and expansion stages of damage. Loutas [18] et al. conducted tensile tests on braided carbon/carbon composites, focusing on the influence of the manufacturing process on the fiber/matrix interface, and conducted online acoustic emission monitoring. The results show that the impact is related to the damage mechanism under different load levels. Bourchak et al. [19] conducted tests on carbon fiber reinforced plastic composite laminates under static and fatigue loads, and used acoustic emission for monitoring. The results show that the AE energy is correlated with the damage observed by ultrasonic scanning and microscope in terms of damage type, location, and effective parameters of damage accumulation. Michalcova et al. [20] used optical equipment and acoustic emission technology to monitor the crack growth of a double-cantilever spar with plain carbon fiber reinforced composite materials in the environmental laboratory, and studied the relationship between acoustic emission energy and damage degree. The results show that the emission energy decreases when the temperature increases and the crack grows.

In addition to damage evolution research, acoustic emission technology is also widely used in wind power blade damage monitoring. Xu et al. [21] used acoustic emission analysis technology to monitor the fatigue test of 59.5 m long blades, extracted the original AE signal features through wavelet packet decomposition, identified the damage mode, and verified the robustness of the method. Tang et al. [22] conducted damage monitoring for the fatigue test of 45.7 m long blades based on AE technology, installed AE sensors near the web and trailing edge, respectively, and introduced initial defects. With the increase in fatigue test times, the growth of fatigue damage caused by primary defects and the location of damage were successfully detected. Zhou Bo et al. [23] used the blind deconvolution separation method to extract the features of acoustic emission signals collected in the fatigue test of 3.95 m long reduced ratio blades, and obtained the characteristics of weak cracks in blade skin and their changing trend with fatigue load. The damage evolution of composite materials is a complex mechanical process with highly nonlinear, multi-excitation source characteristic coupling and a large number of random factors. Different defects have significantly different AE signal characteristics, components, and damage modes in the process of damage evolution, so the physical meaning expressed by their damage modes is different [24]. Therefore, as a necessary prerequisite for the realization of wind power blade health monitoring and condition assessment, it is necessary to clarify the acoustic emission signal characteristics, damage modes, and damage mechanisms of different defects in the damage evolution process, so as to improve the interpretation of defect damage behavior.

In summary, the study of acoustic emission signal characteristics and damage mechanisms of different defective main spar composite materials during the damage process can provide support for the realization of wind turbine blade condition monitoring, contribute to the realization of wind turbine blade health condition monitoring, reduce the incidence of major safety accidents, and reduce the huge economic losses caused by late, untimely repair. In this paper, the artificial prefabrication of GFRP composites containing defects was carried out to solve the most harmful lamination and wrinkle defects in wind power blades. The damage evolution of GFRP laminates with lamination and wrinkle defects was studied using the acoustic emission monitoring technique. The influence of the size and position of different defects on the mechanical properties of materials was analyzed, and the acoustic emission signal characteristics and damage modes of different defects during the damage process were obtained by means of acoustic emission sensor signals, and the damage mechanism of different defects was analyzed by combining the acoustic emission signal characteristics and microscopic results during the damage process. This work has important reference significance for structural damage monitoring and damage evolution mechanism research of wind power blade composites.

## 2. Experiment Methods

### 2.1. Specimen Preparation

Because the thickness of the main spar of the wind turbine blade is much smaller than the plane size of the blade, the change in surface bending deformation can be ignored. Moreover, the normal deflection of the main spar surface is constant along its thickness direction, and the stress perpendicular to the plane direction of the main spar can be ignored. Therefore, the main spar structure of the blade conforms to the applicable conditions of the laminate theory, and the use of GFRP laminate specimens in the study conforms to the practice. According to GB/T25383-2010 wind turbine blade quality standards, wind turbine blades should be tested for process defects during processing and completion, such as wrinkle defects, delamination defects, air bubble defects, lack glue, etc. It is necessary to increase the strength check especially for delamination defects and wrinkle defects, which shows that delamination defects and wrinkle defects are important factors affecting the production quality of wind turbine blades [25].

**Figure 3:**
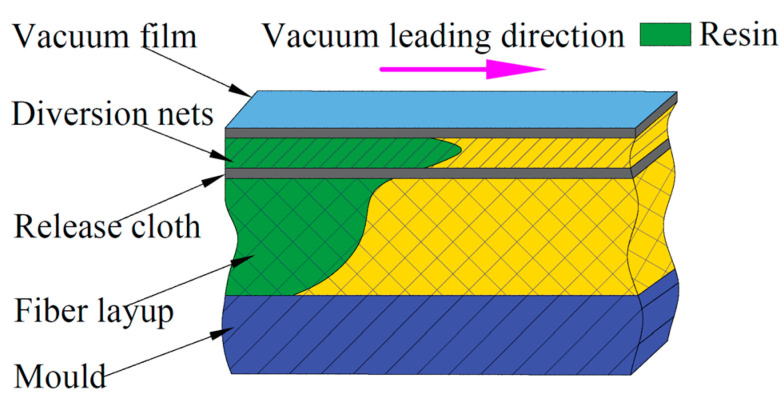
Diagram of resin introduction layer structure.

Vacuum-Assisted Resin Infusion (VARI) was used to make laminates with defective blades. The technique was Resin Transfer Molding (RTM), which is a high-performance and low-cost vacuum-assisted perfusion method developed on the basis of RTM [26]. At present, VARI has become one of the main manufacturing processes for wind power blades, and the manufacturing method is shown in Figure 3. Under the same process conditions, the strength, stiffness, hardness, and other physical properties of the parts obtained by the vacuum perfusion method will be increased by 30–50% compared with the traditional hand paste process, and the porosity can be reduced to less than 0.2%, and the cost can be saved by 50%. In addition, compared with the traditional resin molding method, the vacuum perfusion method can reduce the porosity of composite parts and save 50–70% of the mold cost [27].

**Figure 4:**
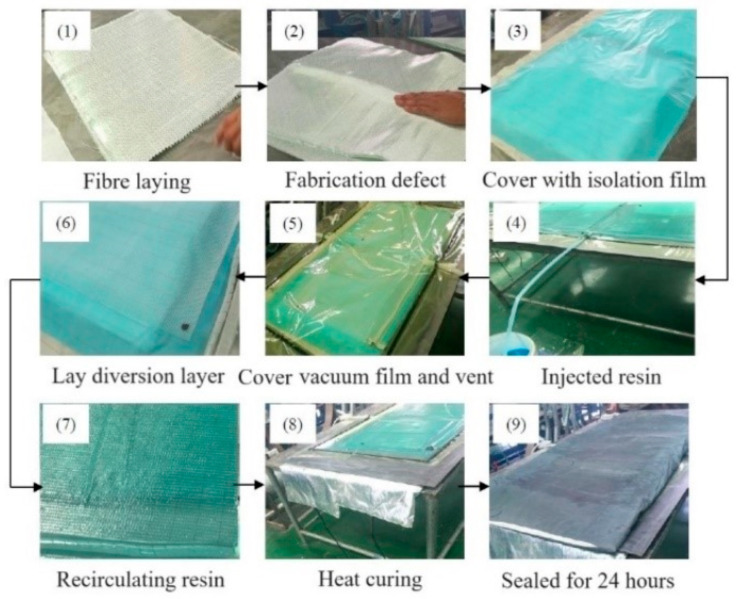
GFRP composite manufacturing process.

Lay a pre-prepared fiber layer on the bottom mold, cover the mold plate after laying, and ensure that the bottom and upper mold can be fully closed. A vacuum bag is used to wrap and seal, and then a vacuum pump is used to pump air to negative pressure. Finally, the resin liquid is injected into the whole mold through the grease injection port, and the resin is fully permeated through the layer structure. The concrete implementation steps of the specimen are shown in Figure 4. Glass fiber unidirectional cloth (ECW600-1270, 600 g/m^2^) is selected as the lay-up material. The epoxy resin used in the preparation process of GFRP composites is Araldite LY 1564 SP, with a density of 1.1~1.2g/cm^3^ and a concentration of 100% transparent liquid. The curing agent model is Aradur 3486, the density is 0.94~0.95 g/cm^3^, and the concentration is 100% blue liquid. The mass ratio of the epoxy resin and curing agent in the resin matrix is 100:34.

Since epoxy resin is a thermosetting resin, the resin liquid and curing agent should be fully stirred and mixed before heating in the preparation process. It is worth noting that in the curing heating process, the temperature cannot be set too high, as too high temperature heating will lead to changes in material properties. At the same time, too low temperature control will lead to poor fluidity of the resin liquid and difficulty penetrating the fiber layer structure. In addition, too low a temperature is not conducive to the removal of bubbles in the resin, and the remaining bubbles will lead to an increase in porosity. After repeated experiments, the curing heating temperature should be controlled at 70~80 °C.

The prefabrication method of artificial lamination defects is made by placing polytetrafluoroethylene film in the fiber prefabrication block. In the process of high temperature curing, the polytetrafluoroethylene film is deposited on the surface of the resin base, resulting in a better fusion between the resin base infiltrated on the surface and the fiber layer, thus forming a weak interface. In the cooling process, the weak interface is separated due to the different thermal expansion coefficient between the polytetrafluoroethylene film (PTFE) and the composite material, so as to simulate the delamination defect. The specific operation process of the artificial layered defect production method in this paper is as follows:

(1). First lay the bottom glass plate mold and keep it clean, and lay the layer from the bottom to the top.
(2). When the delamination operation is carried out to the layered defect design layer, place the PTFE film with a thickness of 0.5 mm.
(3). After the layer is finished, lay the stripping cloth, diversion net, and vacuum bag.
(4). Connect the covered paving structure to the vacuum resin filling system, and check the air tightness of the filling device and mold.

**Figure 5:**
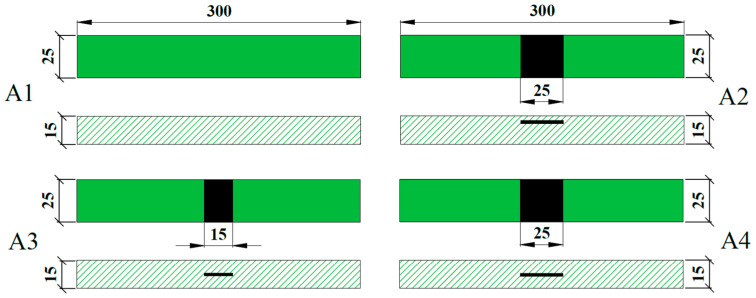
Size diagram of delamination defect specimens (units: mm).

After the inspection is qualified, the grease is injected and finally the curing operation is carried out. The schematic material samples of the four combinations are shown in Figure 5. Specimen A1 has no delamination defect and specimen A2 has PTFE film with a size of 25 mm × 25 mm sandwiched between the third and fourth layers. The PTFE films are 25 mm × 15 mm and 25 mm × 25 mm in size, respectively, sandwiched between the 12th and 13th layers of specimens A3 and A4.

**Figure 6:**
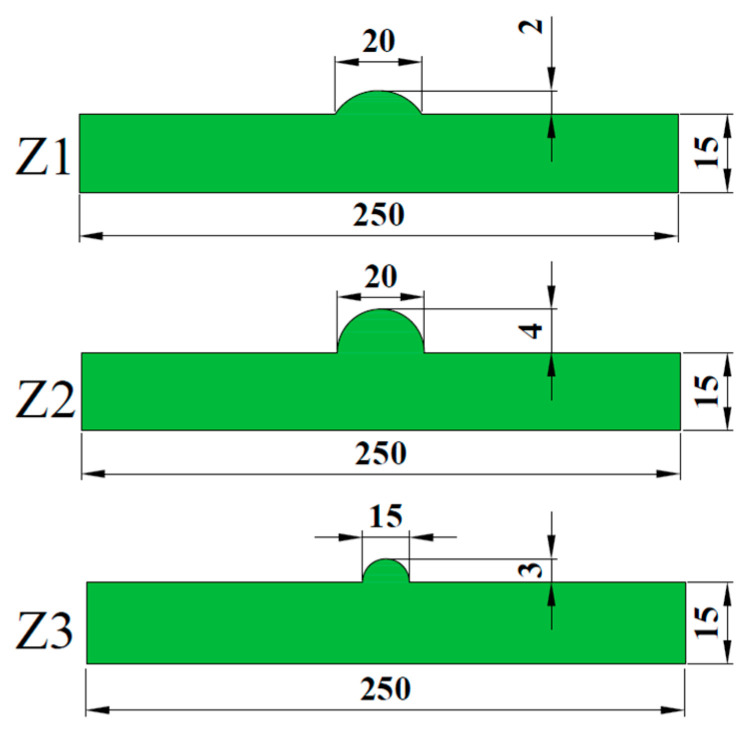
Size diagram of wrinkle defect specimens (units: mm).

When the wrinkle defect is prefabricated manually, the copper wire of uniform material is generally placed between the fiber cloth and the mold. In the process of vacuum perfusion, the pre-placed copper wire can change the angle of the fiber cloth layer and prevent the infiltration of resin on the wrinkle area of the fiber cloth. After cooling and curing, the pre-placed copper wire is removed by electric winch traction, and the prepared resin is injected into the empty slot of the copper wire for re-curing, so that the area is rich in resin, thus simulating the wrinkle defect. In this paper, copper wire with a diameter of φ = 2 mm is used to make wrinkles when preforming wrinkle defects. The specific operation process is as follows:

(1). First clean the sign of the mold plate, and then evenly apply the release agent to the surface of the mold plate.
(2). Place the copper wire with a diameter of *φ* = 2 mm on the mold plate and place the glass fiber cloth on the copper wire, and perform the lay-up operation from the bottom to the top. The copper wire placed at different layer positions produces different aspect ratios, which are, respectively, labeled Z1 (2:0.2), Z2 (5:1), and Z3 (1.5:0.3). The specific size parameters are shown in Figure 6. About 1 m of copper wire is reserved during operation to facilitate fixing on the electric winch.
(3). After the layer is finished, lay the stripping cloth, diversion net, and vacuum bag.
(4). Use an electric winch to remove the copper wire and fill the copper wire empty tank with pre-stirred resin liquid for curing operation. This area is a rich resin area.
(5). Cut the prepared GFRP composite material into a laminate with a predetermined size, polish the gap flat, and finally paste reinforced aluminum sheets at both ends of the specimen.

**Figure 7:**
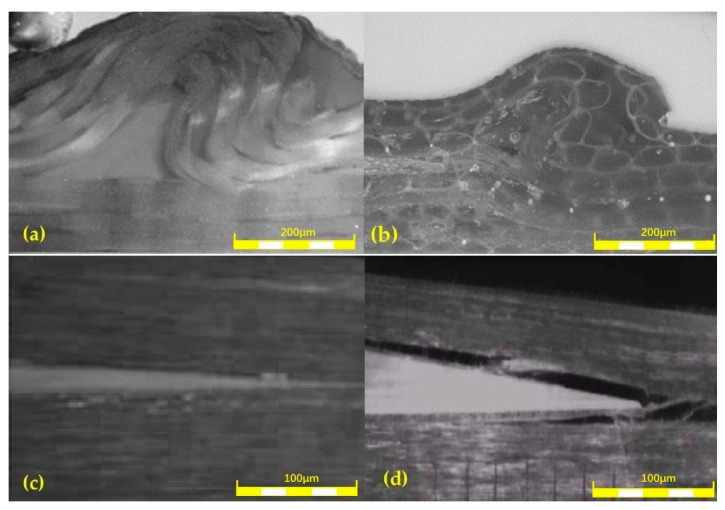
Morphological features of defects: (**a**) artificial wrinkle defects; (**b**) natural wrinkle defects; (**c**) artificial delamination defects; (**d**) natural delamination defects.

The microscopic morphology of the resin aggregation layer with artificial prefabricated wrinkle defects is shown in Figure 7a. It can be seen that the boundary between the resin aggregation area of artificial wrinkle defects and the composite material forms an “Arched” transition zone. The change in the fiber layering angle in this region makes the composite susceptible to stress concentration interference during loading. As shown in Figure 7b after the natural wrinkle defect is polished, the fiber structure near the boundary of the resin gathering area changes, which is consistent with the interface structure at the artificial prefabricated wrinkle defect. In Figure 7c, it can be seen that the fibers near the layered interface at the macro level do not fully fuse with the resin matrix due to the presence of the PTFE film. Natural stratification easily weakens the adhesion of the material interface, resulting in the destruction of the interface structure, and its microscopic morphology is shown in Figure 7d. By comparing the defect morphology of artificial lamination and natural lamination, it can be seen that the artificial lamination defect composite method adopted in this paper can simulate the natural lamination defect better.

### 2.2. Acoustic Emission Test Method

**Figure 8:**
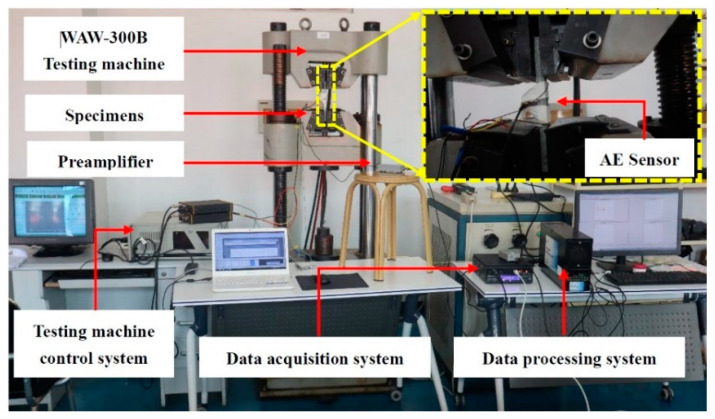
Acoustic emission testing system.

The tensile tests of composites were performed according to the ASTM standard D3039 partly (Standard Test Method for tensile properties of Ploymer Matrix Composite Materials) [28]. The tensile test was carried out on a WAW-300B servo universal test machine. The maximum test force is 300 kN, the relative error of the test force indication value is ≤±1% of the value, and the test force measurement range is 2~100% of the maximum test force. The control range of constant displacement is 0.01 mm/s, and the test system is shown in Figure 8. An acoustic emission sensor was installed at the defect of the specimen and fixed with tape. In order to provide a good acoustic coupling between the specimen surface and the sensor, silicone grease was used as the coupling agent and a pencil fracture test was performed. In order to ensure the effective elimination of electrical and mechanical noise, the appropriate threshold is fixed at 40 dB through repeated testing. The initial load is applied to eliminate the noise interference in the loading process of the reinforced aluminum sheet.

**Figure 9:**
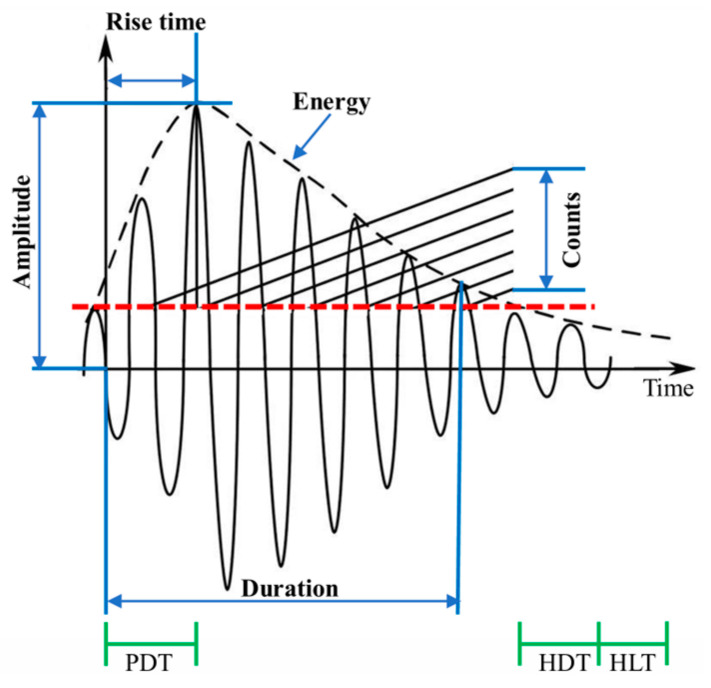
Definition of acoustic emission parameters.

It is worth noting that the quality of acoustic emission data obtained by the experiment mainly depends on the timing parameters of the time-domain waveform. The PDT (peak definition time), HDT (hit definition time), and HLT (hit lock time) are parameters used to select acoustic emission event characteristics. The main acoustic emission parameters and definition methods obtained in the experiment are shown in Figure 9. In the experiment, the center frequency of the acoustic emission sensor is 150 kHz and the gain of the preamplifier is 40 dB. The sampling frequency is set to 3 MHz, the peak definition time is 30 μs, the peak definition time is 150 μs, the hit definition time is 150 μs, and the hit lock time is 300 μs [29].

**Figure 10:**
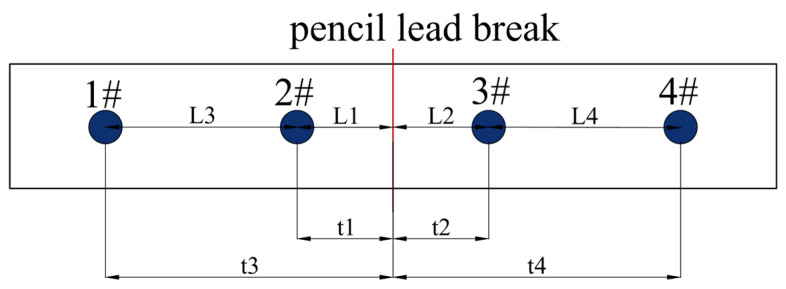
Sound velocity calibration for acoustic emission lead break test.

The sensor selects four symmetrical sensor channels, and the position of the sound source is the real lead break position, that is, the red line position, as shown in Figure 10. Through the arrival time of the acoustic emission signal received by the sensor and the distance of the sensor ruler, the calibration of the acoustic emission wave speed is completed. Considering the complex structure of the composite material itself, the acoustic velocity calibration of the acoustic emission signal needs to be measured several times, and the average value is taken. In order to solve the attenuation problem of acoustic emission signals, the sensor signals at different positions are tested during the lead-breaking test. The amplitude of the lead-breaking signal collected by the sensor should not be less than two times of the threshold amplitude. Due to the close distance between the acoustic emission sensor and the defect position in this paper, the attenuation effect of the acoustic emission signal is relatively small. The sound velocity calculation method is shown in Equation (1).

(1) $$\bar{V}=\left(\frac{L_{1}}{t_{1}}+\frac{L_{2}}{t_{2}}+\frac{L_{3}}{t_{3}-t_{1}}+\frac{L_{4}}{t_{4}-t_{2}}\right)/4$$

In the lead-breaking experiment, a 0.5 mm pencil was used, the lead length was about 2.5 mm, and the angle between the lead and the specimen surface was about 30°. The signal frequency of the experimental interrupt lead test is distributed in 100–400 kHz. When the characteristic frequency of the broken lead signal is 150 kHz, the amplitude of the signal is the largest, and the other low amplitude signals may be the interference of environmental noise.

In the test process, the number of sensors is first determined by the attenuation of the pencil lead-breaking signal in the pre-test. The specific operation method is as follows: Two AE sensors, 40 mm apart, were arranged on the top and bottom of the laminate specimen, and a pencil lead-breaking test was carried out at the defect center. The AE wave velocity and attenuation amplitude of the laminates were 4423.7 m/s and 0.22~0.38 dB/cm, respectively, and the maximum time difference of AE signal attenuation in the pencil lead-breaking test was determined (Δ*tmax* = 0.0084 ms).

**Figure 11:**
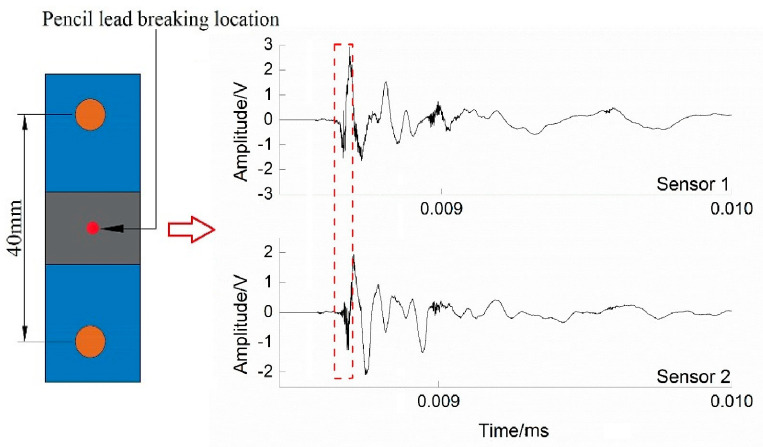
The schematic of pencil lead break test.

An acoustic emission lead-breaking waveform is shown in Figure 11. As can be seen from the figure, the time difference between sensor 1 and sensor 2 to receive the acoustic emission signal of broken lead is 0.0018 ms (<Δ*tmax* = 0.0084 ms). The test results show that both sensors can effectively receive acoustic emission signals, and there is no significant difference in signal attenuation. Therefore, the use of an acoustic emission sensor in the experiment can also effectively capture the damage characteristics, which is consistent with the experimental conclusion of Favretto [30].

## 3. Experimental Results

### 3.1. K-Means Clustering Analysis of AE Signals

The acoustic emission waveform is stored by a computer and acquisition system, and the corresponding characteristic parameters such as energy, amplitude, and count are calculated according to the acoustic emission waveform. Since these characteristic parameters are derived from acoustic emission acquisition waveforms, the damage characteristics of different defects can be obtained by analyzing them to a certain extent. The acoustic emission characteristic parameters obtained in the experiment mainly include: amplitude, energy, count, and peak frequency, etc. All acoustic emission characteristic parameters are regularized [0, 1], and the characteristic parameter matrix is established. Due to the complexity of composite structure, the AE source has diversity and uncertainty, and different AE sources can produce completely different AE signals. However, in real situations, these feature parameters may exhibit high similarity. This is because they are all different ways of quantifying physical phenomena produced by the same acoustic emission source, and these physical phenomena are inherently related to each other. A fully linked geometric distance metric was used to cluster the feature parameters, and the results show that the AE number, energy, peak frequency, and amplitude height have some similarity in describing the damage trend, as shown in Figure 10. Therefore, the feature parameters with high similarity are selected for K-means clustering analysis.

**Figure 12:**
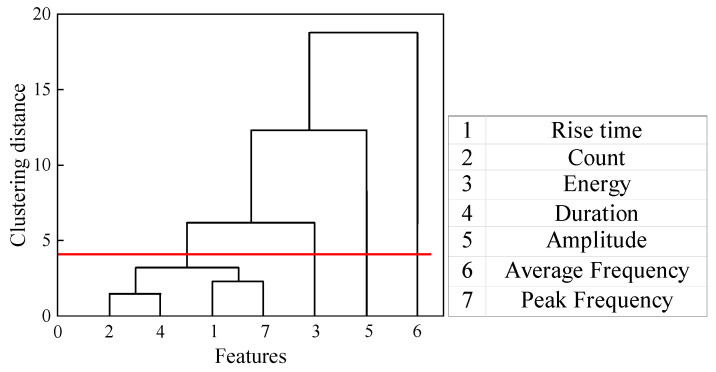
Correlation tree diagram of acoustic emission signal parameters.

The K-means clustering algorithm classifies the data features in the sample space, and uses the optimization iteration function to correct the clustering results in the initial supervised learning sample. The K-means clustering algorithm is a clustering algorithm based on the optimal criterion function, and the criterion function is as follows [31]:

(2) $$J=\sum_{i=1}^{k}\sum_{x\epsilon C_{i}}{\left\|x-m_{i}\right\|}^{2}$$

where *x* is the acoustic emission parameter data sample, *k* is the number of clusters, *C_i_* is the sample set of the Class *i* cluster, *m_i_* is the mean vector of cluster *i*, and satisfies the following:

(3) $$m_{i}=\frac{1}{n}\sum_{x\in x_{i}}x_{i}$$

where *n_i_* is the number of samples in *C_i_*. Criterion function *J* is used to describe the total error square generated by the *k* cluster sample set *C*_1_, *C*_2_, ···, *C_k_* represented by different clustering centers *m*_1_, *m*_2_, ···, *m_k_*. The smaller the *J* value, the higher the similarity of data in the same cluster sample set. The K-means clustering algorithm uses the iterative function to continuously adjust the clustering center, so that the error sum of squares criterion function **J** of each cluster sample set can obtain the minimum value. Therefore, the number of clusters *k* and the initial cluster center should be determined before using this method, as shown in Figure 12.

Due to the high sensitivity of acoustic emission sensors to weak signals, it is also susceptible to noise interference. During the experiment, the influence of environmental noise should be avoided as much as possible, but the noise source of the experimental equipment itself cannot be avoided. The AE data obtained at a high sampling frequency are very large, so principal component analysis (PCA) is adopted to preprocess the AE features, reduce the information dimension, and screen effective AE parameters.

The comatrix is established after the normalization of the acoustic emission characteristic parameters selected in the experiment. *C* is set to represent the comatrix of acoustic emission characteristic data. The eigenvalue *λ*_i_ can be calculated by the equation *det*(*C* − *λ*_i_) = 0. The columns of the eigenvector matrix *A* satisfy the equation *C* = *ADA^T^*, where the matrix *D* is the eigenvalue *λ*_1_, *λ*_2_, …, *λ*_m_ forming a diagonal matrix, and *λ*_1_ ≥ *λ*_2_ ≥ … ≥ *λ*_m_. PCA can transform multidimensional AE data into a series of linearly uncorrelated new features called principal components. Let *A* be a matrix of eigenvectors, *a_ij_* is the element that makes up *A*, and then each principal component can be represented as follows:

(4) $${Pd}_{i}=\sum a_{ij}\bar{p_{j}}$$

where $\bar{p_{j}}$ is the initial set of normalized AE signal parameters. The AE signals after PCA dimensionality reduction were analyzed by K-means clustering. The number of clusters was evaluated using the Davis–Bouldin and Silhouette indices, and the higher the *DB* index and the lower the *SI* index, the best number of clusters for the sample data [32,33].

(5) $$DB(k)=\frac{1}{k}\sum_{i=1}^{k}max(\frac{{DW}_{i}+{DW}_{j}}{{DC}_{ij}})$$

(6) $$SI=\frac{1}{n}\sum_{i=1}^{n}\frac{b\left(x_{i}\right)-a\left(x_{i}\right)}{max\left\{a\left(x_{i}\right),b\left(x_{i}\right)\right\}}$$

where *DW_i_* represents the average distance of the center of mass between adjacent clusters. *DW_j_* represents the average distance of the center of mass between the cluster center and other clusters. (*DW_i_ + DW_j_*)/*DC_ij_* represents the similarity between different clusters, which is the clustering distance. *a*(*x_i_*) represents the average distance between *x_i_* and other clusters, and *b*(*x_i_*) represents the average distance between *x_i_* and neighboring clusters.

**Figure 13:**
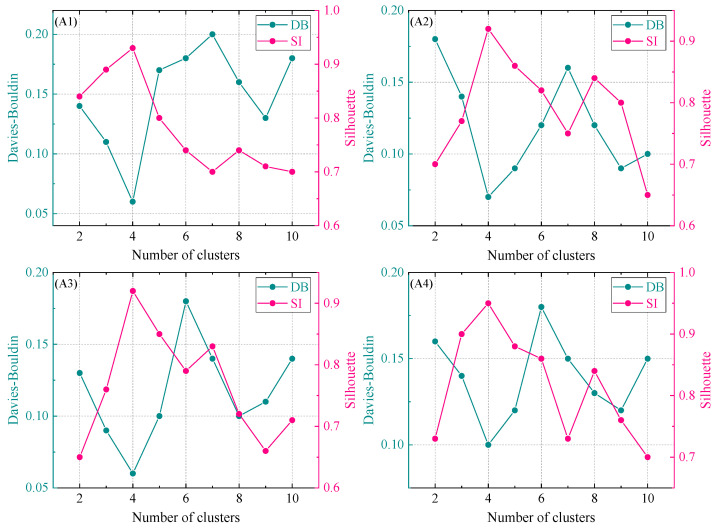
Evaluation of the number of clusters: specimens A1, A2, A3, and A4.

Reference [34] compared the clustering sensitivity of different acoustic emission parameters. The results show that the acoustic emission amplitude, average frequency, and energy have high variance values, which means that these three acoustic emission parameters will obtain better clustering results. The clustering number results of composite specimens are shown in Figure 13. It can be seen from the evaluation results of the *DB* and *SI* index that the optimal cluster number is 4.

The clustering results and AE signal quantity of samples with delamination defects and wrinkle defects are shown in Table 1 and Table 2. The matrix cracking stage is represented by Cluster 1’s characteristic center frequency of 63.2–65.8 kHz, the fiber/matrix stripping stage by Cluster 2’s characteristic center frequency of 136.9–142.87 kHz, the delamination of the interface stage by Cluster 3’s characteristic center frequency of 254.46–284.3 kHz, and the fiber breakage stage by Cluster 4’s characteristic center frequency of 365.2–397.75 kHz, according to the clustering results of delamination defect specimens. The typical center frequencies of Cluster1 matrix cracking and Cluster2 fiber/matrix stripping were 69.38–73.22 kHz and 137.24–148.53 kHz, respectively, in the clustering results of wrinkle defect specimens. The typical center frequency of Cluster4 fiber breakage is 392.83–402.27 kHz, while the characteristic center frequency of Cluster3 interface delamination is 281.61–287.20 kHz, which corresponds to the interface delamination stage.

In addition, the low frequency component whose peak frequency is less than 30 kHz is due to mechanical vibration interference during loading. The amplitude of acoustic emission signals can represent the intensity of sound source features, and the amplitude distribution is related to the deformation mechanism inside the material. According to Table 1 and Table 2, during the damage evolution process, glass fiber composites containing lamination defects and wrinkle defects will generate a large number of high-amplitude AE signals. In the initial stage of damage evolution, the matrix cracking and fiber/matrix stripping damage characteristics of layered defect specimens are more active than those of non-defective specimens. The reason is that the existence of delamination defects will affect the strength of the interlayer structure, leading to the rapid deterioration of matrix cracking and fiber/matrix spalling during the tensile process. By comparing the defect specimen with the no-defect specimen, it can be found that the damage characteristics of the layered defect and the wrinkle defect are basically the same. However, the central frequency of the damage mode characteristics is different, which indicates that the composite with defects has a certain influence on the damage characteristic frequency and produces fluctuations.

**Figure 14:**
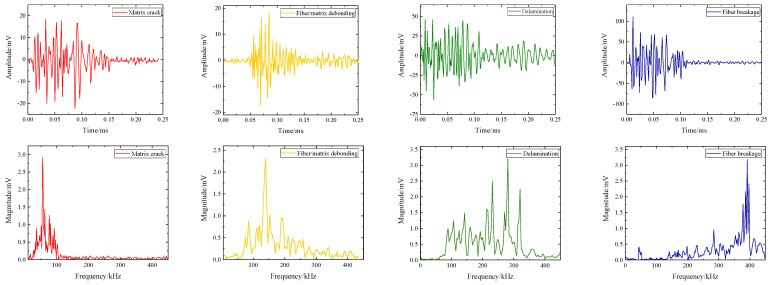
Time domain and frequency domain signals of different damage modes.

As shown in Figure 14, acoustic emission waveforms and spectrum characteristics of different damage modes were extracted according to the clustering results. The damage characteristic spectrum of real wind turbine blades and the acoustic emission waveform characteristics of glass/epoxy composites were compared. By comparing the damage pattern recognition results of the reference and this paper, the characteristic frequency range of the four damage patterns is basically consistent with the reference results. The time-domain waveform duration of high-frequency signals is shorter, and the time-domain waveform duration of low-frequency signals is longer [35,36,37]. It is worth noting that the acoustic emission signals of matrix cracks in real wind turbine blades in reference [36] are compared. In this paper, the acoustic emission signal amplitude representing the low-frequency matrix crack fluctuates greatly, as shown in Table 1. The reason for this phenomenon is that in the damage process, the matrix usually starts with small cracks and gradually expands into larger cracks, which leads to obvious amplitude fluctuation. In addition, due to the influence of defects, the matrix structure is more likely to produce high-amplitude acoustic emission characteristics during the damage process.

**Figure 15:**
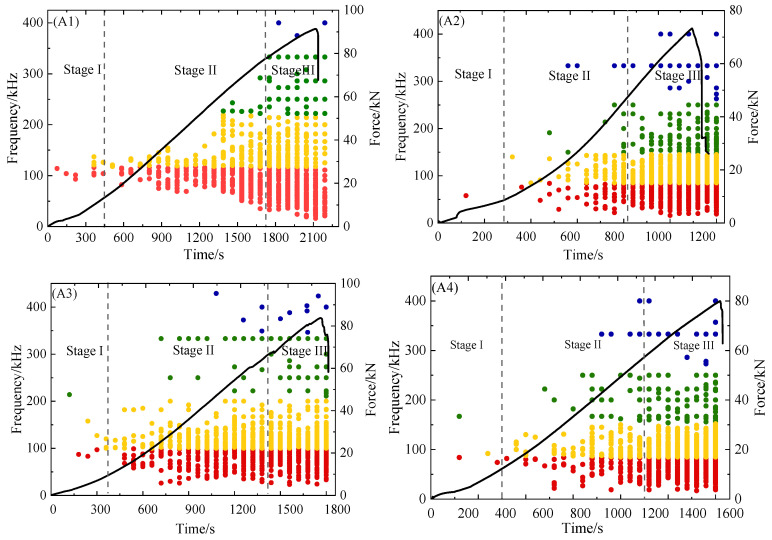
Frequency and load changes with time for different specimens.

The relationship between tensile load and peak frequency and time is shown in Figure 15. The grouping boundaries of Cluster 1 and Cluster 2 AE signals in the low frequency stage have overlap in the peak frequency clustering results. This behavior is caused by the low frequency stage’s peak amplitude being very modest and the signal there being highly contaminated by noise. Therefore, the data partitioning accuracy in the process of cluster analysis will be affected [38]. It can be seen from Figure 15 that in the damage mode of the low frequency stage, the entire stretching process happens virtually concurrently with matrix cracking and fiber stripping. Due to the constant evolution and quick spread of faults, many fiber breakage characteristic frequency signals emerge prior to reaching the fault point. The findings indicate that four damage modes will manifest simultaneously and a large number of high-frequency signals will manifest when the composite is close to failure.

According to the results of clustering in Table 1, AE signals of fiber bundle breakage are characterized by peak frequency and low amplitude, and samples with delamination defects generate AE signals of peak frequency earlier than standard specimens. In the second stage of damage evolution, fiber/matrix stripping and propagation lead to the continuous emergence of high-frequency AE signals. For sample A1 shown in Figure 15 (A1), AE signals with high-frequency fiber breakage began to appear when the load was increased to about 80 kN. Similarly, for specimens A2, A3, and A4 with delamination defects in Figure 15 (A2–A4) with AE characteristics of high-frequency fiber breakage, the corresponding loads were 45 kN, 75 kN, and 55 kN, respectively. Compared with specimens A1, A3, and A4, the high-frequency fiber breakage feature of specimen A2 appears at about 550 s, which is earlier than the peak frequency and irreversible damage time of other specimens. This is because the closer the lamination defect is to the surface of the specimen, the lower the structural bearing capacity of the specimen.

According to the above analysis, defect depth and size are related to AE characteristic frequency. As shown in Figure 15, the depth and size of defects in the damage evolution process of GFRP composites have a certain influence on the frequency characteristics of fiber breakage. In layered defects, when the defect depth is the same, the characteristic frequency of the fiber breakage of the specimen with a large defect size is smaller than that of the specimen with a small defect size. When the defect size is the same, the fracture characteristic frequency of the defect depth in the surface layer is smaller than that in the middle layer. Similarly, the effect of wrinkle defects with different aspect ratios on damage patterns is roughly the same as that of layered defects. Therefore, the frequency characteristics of the damage mode of composite materials are related to the physical properties of the material itself, but different defect sizes and depths will affect the frequency range of the damage mode characteristics.

**Figure 16:**
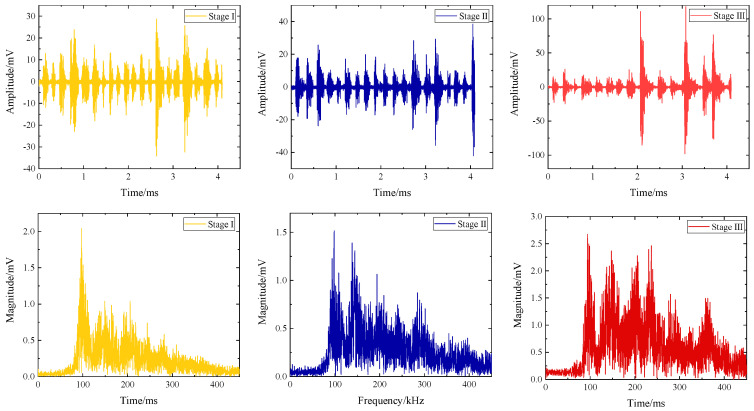
Acoustic emission signal spectrum characteristics of specimen A1 at different damage stages.

As shown in Figure 16, the peak frequency of acoustic emission signals in damage stage I is 93 kHz, and the frequency components in this stage are mainly low-frequency characteristics, and there are almost no high-frequency fiber breakage components larger than 350 kHz. In stage II, the acoustic emission peak frequency is mainly 93 kHz, 150 kHz, and 210 kHz, and there is a frequency characteristic of 290 kHz. In this stage, the main damage is matrix cracking, fiber breakage, and interface delamination. In stage III, in addition to the low and medium frequency features, a high-frequency feature with a peak frequency of 378 kHz appears, indicating that the damage component of fiber breakage is very active in this stage. The spectrum characteristics of the above transmitted signals are basically consistent with the clustering results in Figure 15 (A1).

### 3.2. Acoustic Emission Characteristics of Delamination Defects

**Figure 17:**
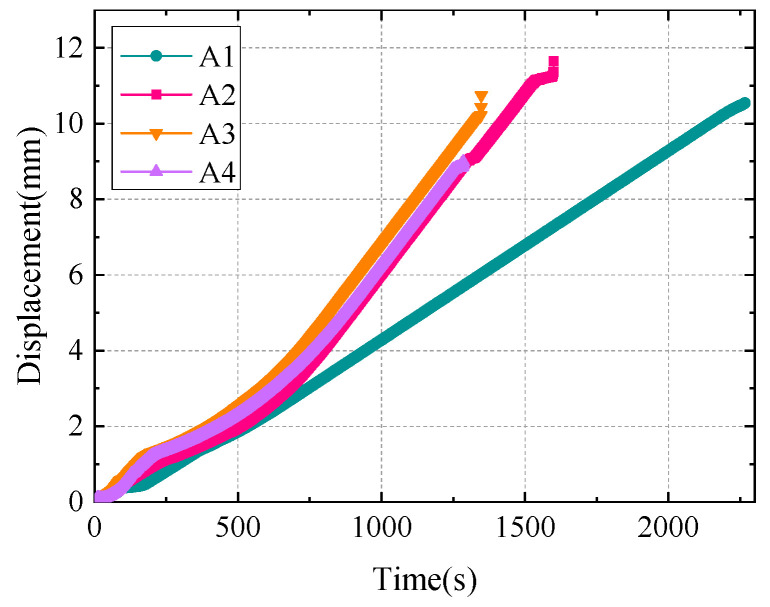
Displacement curve of specimens in tensile test.

According to the tensile test of the composite material specimens with layered defects, the average failure loads of layered specimens A1, A2, A3, and A4 are determined to be 92.8 kN, 76.41 kN, 85.24 kN, and 79.73 kN, respectively. The displacement change trend of the composite material sample in the tensile test is shown in Figure 17. In the experiment, the ultimate breaking load of specimens A1, A2, A3, and A4 is significantly lower than that of specimen A1 when the layered defects are present. This shows that lamination defects can reduce the ultimate strength of composite structures, and the closer the lamination defects are to the surface of composite materials, the greater the impact on the structural strength of composite materials. In addition, when the location of the lamination defect is the same, the larger the size, the greater the influence on the structural strength of the composite.

**Figure 18:**
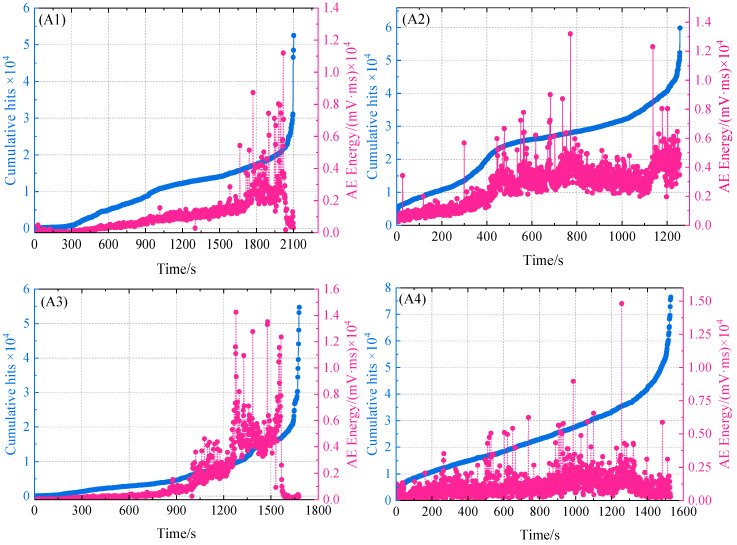
AE accumulation energy and accumulation count distribution over time: specimens A1, A2, A3, and A4.

AE amplitude, accumulation count, energy, and count parameters were selected as AE feature analysis in the tensile test results. Figure 18 displayed the correlation between the specimen’s accumulated count variations over time and the AE accumulation energy. A low energy amplitude change is nevertheless visible in the first phase, despite the load being smaller and the rising rate being slower. This behavior demonstrates that the AE signal gathered at this point is essentially a noise signal, making it vulnerable to interference from the noise signal during the initial loading stage. From specimen A1 in Figure 18 (A1), it can be found that AE energy amplitude fluctuates greatly with the increase in time, which is related to the significant increase in sample damage degree. When t = 1600 s, the cumulative count and energy of the AE signal increase rapidly. This phenomenon shows that a large number of high-energy AE signals appear with the beginning and accumulation of damage in the composite material, which indicates that the early energy accumulation is partially released at this stage, and the irreversible damage evolution begins.

Compared with specimen A1 without delamination defects, the AE characteristics of delamination specimens A2, A3, and A4 shown in Figure 18 (A2–A4) are significantly different. As can be seen from Figure 18 (A2), due to the existence of stratification defects, the AE energy peak and accumulation count of specimen A2 showed an obvious upward trend when t = 400 s. The reason is that the delamination defect has a great impact on the structural strength of the material, which directly leads to the irreversible damage caused by the delamination defect earlier in the damage process. By comparing specimens A2 and A4, it can be seen from Figure 18 (A4). When the depth of defect increases, the peak value of AE energy and the active trend of the accumulation count during the damage process decrease to a certain extent. The results show that when the size of the lamination defect is the same, the closer the position of the defect is to the surface of the specimen, the greater the influence on the structural strength of the main spar composite.

It can be seen from Figure 18 (A3) that when t = 1050 s, the AE energy peak and accumulation count of specimen A3 show an obvious upward trend, and the activity of the AE characteristics of specimen A3 in the stable damage stage is significantly weaker than that of specimens A2 and A4. By comparing the emission energy peak and accumulation count of specimens A2, A3, and A4, it can be seen that the size of layered defects has the greatest influence on the damage evolution of materials, followed by the depth of defects. The existence of delamination defects in the damage process of the material will lead to the damage of the fiber layer, and the defect part will be damaged in advance under the action of a high strength load. Furthermore, the entire fiber sliding off at the bridging contact, the stripping of the fiber–matrix interface, and the damaged defects influence the surrounding matrix tissue. High-energy AE signals are produced as the fibers break and pull out, reducing the structural strength within the layer. This can be explained by the significant quantity of energy released when the specimen’s internal structural damage rapidly deteriorates.

**Figure 19:**
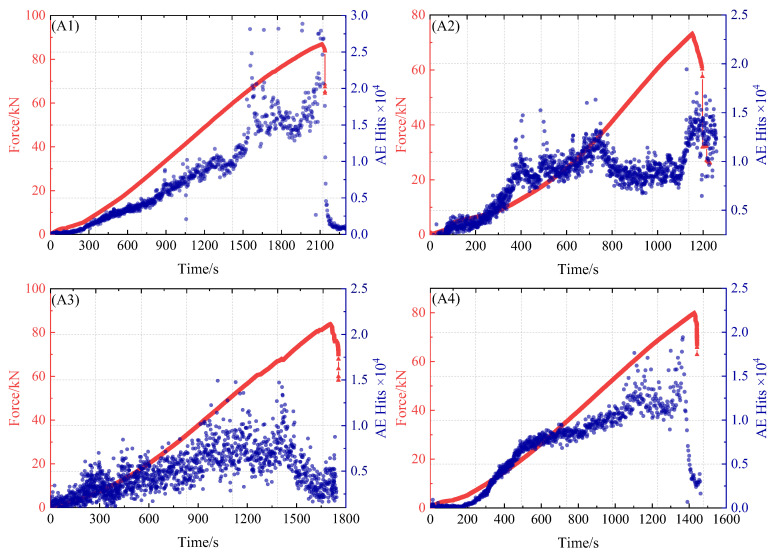
Tensile force and acoustic emission impact history of delamination defect specimens: specimens A1, A2, A3, and A4.

According to the load curve of layered defective specimens and the AE impact count history diagram in Figure 19, it can be seen that the AE impact count history diagram in the damage evolution process can be divided into three stages: (1) In the first stage, although a small amount of acoustic emission impact number was generated in the initial stage of loading, its fluctuation range was small. According to the analysis of acoustic emission frequency variation in the loading process in Figure 15, it can be seen that the matrix structure of the composite material will produce random distributed micro-cracks and sporadic fiber breakages at the initial loading stage, resulting in the corresponding acoustic emission signals. However, such damage has little effect on the overall stiffness of the specimen, so the acoustic emission impact count presents a gentle linear increase. (2) In the second stage, with the increase in load, the internal structural damage of the composite material is aggravated, and the acoustic emission count increases rapidly at this time. Due to the influence of the stress state of the fiber and the interface, the fiber bundle tends to stretch and rotate in the direction of stretching. At this time, the matrix is subjected not only to tensile stress, but also to extrusion stress. Under the combined action of these stresses, the matrix presents plastic shear failure. This destruction reduces the bearing capacity of the material structure to some extent, resulting in an active AE accumulation count. (3) In the third stage, due to the tensile stress and shear stress of the fiber, the fiber breakage and fiber stripping damage are very severe. Therefore, the acoustic emission signal increases rapidly and reaches the maximum amplitude. Most of the acoustic emission signals in the whole loading process come from the third stage, which is consistent with the mechanical analysis of the specimen.

### 3.3. Acoustic Emission Characteristics of Wrinkle Defects

**Figure 20:**
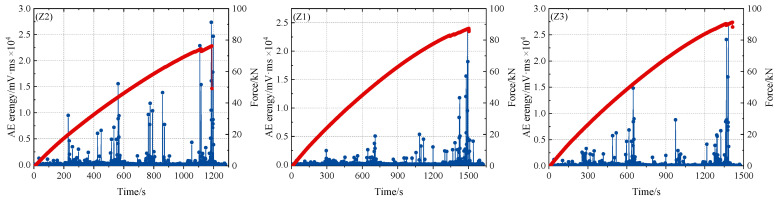
Relationship between load and energy of wrinkle defect.

The aspect ratio parameters of wrinkle defect specimens Z1, Z2, and Z3 are shown in Figure 6. The experimental conditions, parameter settings, and number of sensors in the experimental process are the same as those in the layered defect test method. AE amplitude and energy are selected as AE feature analysis in the tensile test results. The tensile load–AE relative energy curve of wrinkle defect specimens is shown in Figure 18. The fracture failure loads of wrinkle specimens Z1, Z2, and Z3 are 87.6, 76.2, and 82.1 kN, respectively. By comparing Figure 20 (Z1, Z2), when the wrinkle height is the same, wrinkle defects with different aspect ratios have different effects on the mechanical properties of composite material specimens. When the wrinkle aspect ratio is smaller, the mechanical properties of the specimens will decrease more. Compared with Figure 20 (Z2 and Z3), when the wrinkle aspect ratio is the same, the height change in wrinkle defects has different effects on the mechanical properties of composite material specimens. The greater the wrinkle height value, the more the mechanical properties of the specimens decrease.

As can be seen from Figure 18, the overall trend of acoustic emission energy increases with the increase in load. At the early stage of loading, the internal damage of the composite material has no obvious change, and the acoustic emission energy is relatively low. However, with the increase in load, the AE energy shows a gradually increasing trend, which indicates that the damage accumulation and evolution inside the composite material frequently release high-energy characteristics. When the fracture failure load is reached, the instability failure occurs inside the composite structure, and the variation trend of acoustic emission energy reaches a peak.

**Figure 21:**
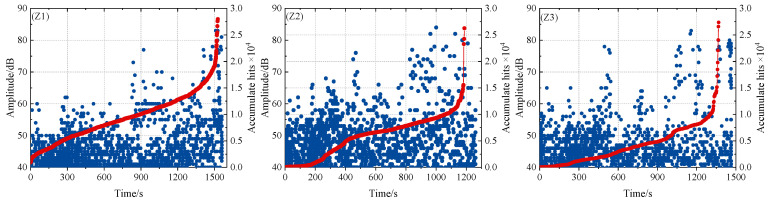
Time history of AE amplitude and accumulated hits of wrinkle defect.

As shown in Figure 21, the acoustic emission amplitude and accumulation count of different wrinkle defect specimens during the whole loading process can be divided into two stages: damage accumulation and damage destruction. In the initial loading stage, there are some high-amplitude signals, but the cumulative count rises slowly, indicating that the AE signals collected by the sensor in the initial stage contain noise signals [39].

In addition, different from single-medium metal materials, the damage evolution of composite materials is a complex random damage process. In the process of damage evolution, random fiber breakage damage characteristics are usually accompanied by higher energy. Therefore, discrete peak values are associated with random fiber breakage damage within the material. Although random fiber breakage occurs during the damage process, a small amount of fiber breakage does not affect the structural strength of the material.

With the increase in load, some acoustic emission signals with an amplitude higher than 60dB appear, which may be related to the evolution of matrix cracking damage in the rich resin region of wrinkle defects. In the damage accumulation stage, the acoustic emission signals are mainly distributed in the amplitude range of 50~65 dB, and the acoustic emission accumulation count increases steadily. During the damage and failure stage, acoustic emission signals with an amplitude ranging from 50 dB to 65 dB increased significantly, indicating that the matrix damage in the specimen structure was aggravated, the accumulation and count of acoustic emission accelerated, and high-amplitude acoustic emission signals greater than 70 dB continuously appeared until the specimen was broken. In addition, by comparing the variation trend of AE characteristics of different wrinkle defects in Figure 21, the AE accumulation and counting of wrinkle specimens Z2 and Z3 in the damage and failure stage are significantly faster than that of wrinkle specimen Z1, which is related to the aspect ratio of wrinkle defects. The smaller the aspect ratio of the wrinkle, the more the mechanical properties of the material decline and the more obvious the structural deformation during the loading process, resulting in more active acoustic emission characteristics in the damage and failure stage.

### 3.4. Damage Mechanism Analysis of Specimens with Different Defects

The count of acoustic emission signals and the statistics of energy parameters in the experimental drawing process are shown in Table 3. The AE amplitude distribution of GFRP composites with different defects during the stretching process is generally between 40 and 90 dB. The AE count increases significantly during matrix cracking and fiber stripping, which is related to the active degree of damage characteristics. Acoustic emission energy characteristics can be seen in the phase of amplitude less than 60 dB, and the energy release is very small. However, with the intensification of damage evolution, the AE energy increases gradually, especially in the high amplitude range, and the emission of AE energy is more active. As shown in Table 3, the AE characteristics of different defect types overlap. Compared with delamination specimen A2 and wrinkle specimen Z1, the AE impact percentage of both is 0.5% in the range of 85~90 dB.

In the whole stretching process, with the increase in load, the proportion of the high-amplitude signal in the acoustic emission signal increases. The acoustic emission characteristics vary significantly with the load in the general trend, especially when the composite material specimen is in the instability and failure stage, and the “crackling” emitted by the specimen can be clearly heard, accompanied by an acoustic emission amplitude higher than 70 dB. In the whole stretching process, the AE counts with an amplitude higher than 70dB account for 7.2~8.8% of the total AE events, and the AE energy accounts for 93.9~96.2%. The reason is that in the uniaxial tensile process of the GFRP composite with a uniaxial lamination structure, the force direction of the structure is consistent with the direction of the fiber. However, the matrix inside the material first succumbed to the force and produced plastic deformation. The existence of glass fiber inhibits the crack expansion of the matrix to a certain extent, and the strain energy inside the material accumulates and releases through the interface between the fiber and the matrix, resulting in interface debonding and delamination, which leads to the weakening of the interface properties of the material. The matrix crack, along with the shear failure, causes a large area of fiber stripping and releases a lot of energy. Due to the particularity of a one-way GFRP-laminated structure, fiber is the main load bearer of the structure, and the matrix mainly plays the role of fixing the fiber and transferring load. Therefore, the fracture failure mechanism of materials is mainly caused by a large number of fiber breakages and longitudinal splitting, rather than interface debonding [40].

Obviously, when the unidirectional GFRP composite is subjected to tensile load, the interface properties of the fiber and matrix are weakened, which are manifested as matrix cracking, fiber stripping, and interface delamination. The experimental analysis shows that during the evolution of the tensile damage of GFRP composites, the acoustic emission characteristics change in different stages, and the intensity, energy, and count of acoustic emission signals are different in each stage. In the initial stage of fiber failure, the amplitude, count, energy, and duration of fiber failure are low, while the AE signal of fiber breakage shows high intensity in the instability stage. The whole test process of the AE signal can be divided into three stages: (1) At the stable stage, the friction and damage evolution degree between the fiber matrix inside the specimen is low, and the acoustic emission activity inside the specimen is weak. (2) During the slow rising period, the internal damage of the specimen began to intensify, evolve, and accumulate gradually, and then rose slowly, mainly because the internal damage of the specimen accumulated to a certain extent and released energy, resulting in small-scale macroscopic damage. (3) During the accelerated rise period, when the stress concentration reaches a certain extent, irreversible damage evolution occurs inside the specimen, and the internal damage increases sharply. AE accumulation and count are characterized by accelerated release, indicating that the damage enters the instability failure expansion.

**Figure 22:**
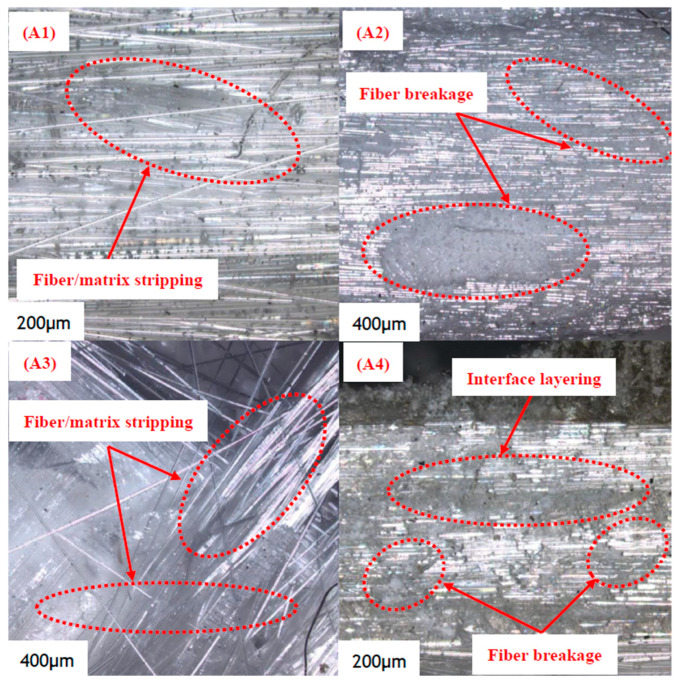
Micrograph of delamination defect specimens after fracture.

The metallographic microscope of the fracture position of the layered defect specimen is shown in Figure 22. The damage mode and failure form of the sample can be clearly observed through a scanning electron microscope. Figure 22(A1) shows the fracture micromorphology of specimen A1, from which fiber stripping and fracture can be clearly seen. Because A1 is a no-defect specimen, the fiber inside the structure has high bonding strength with the matrix, and the stress is evenly distributed along the fiber direction under the action of load. Therefore, there is no obvious bending deformation of glass fiber after sample A1 fracture. Figure 22(A2) is the microtopography of the fracture of the A2 specimen, from which it can be observed that a large area of fiber breakage failure is observed, and some fibers are not sufficiently bonded to the matrix, resulting in the fracture of nearby fibers. Furthermore, a large number of fiber breakages distribute a portion of the load to the adjacent fibers, causing an unequal distribution of stress, as indicated by the random distribution of fiber breakages. Figure 22(A3) shows the fracture micromorphology of the A3 specimen. The fibers separate from the matrix as a result of the interface between the local fibers and the matrix breaking. Due to the existence of delamination defects, the strength of the interlayer structure is reduced, and numerous fibers are pushed out when the matrix’s adherence to the fiber disintegrates. Figure 22(A4) shows the fracture microstructure of A4. Due to the large size of the delamination defect of this specimen, the bearing capacity of the fiber and the binding effect between the fiber and the matrix are the primary determinants of the internal stress distribution of the material under load. The stretching of the fiber bundle itself is primarily responsible for the distortion of the fiber when it starts to experience stress. However, the fiber bundle and matrix will slip and dislocate when the external force is raised further, resulting in severe fiber deformation. At this point, the material structure’s bearing capacity significantly decreases due to large-area fiber breakage and interface delamination, ultimately leading to failure.

**Figure 23:**
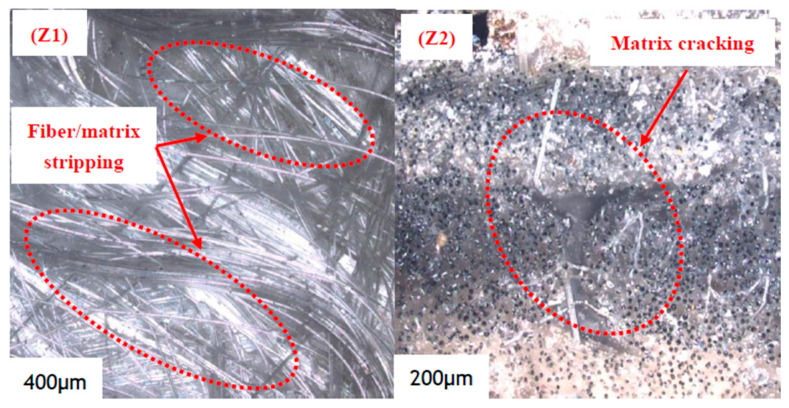
Micrograph of wrinkle defect specimens after fracture.

The metallographic microscope of the fracture position of the wrinkle defect specimen in the experimental results is shown in Figure 23. Different from the layered defects, the fibers are bent at a certain angle due to the existence of wrinkle defects, and the fibers are scattered and accompanied by distortion after the specimen is broken. Fiber breakage occurs at the interlayer of the coated fiber, and interface debonding occurs as a result of the debonding and sliding between the fiber and the matrix, as shown in Figure 23(Z1). Considering the mechanical characteristics of wrinkle defects, the rich resin region needs to bear a combination of axial tensile, compressive, and bending loads under the action of loads [41]. Therefore, it can be seen from Figure 23(Z2) that matrix cracks are more likely to accumulate in the rich resin region of wrinkle defects, and the stress concentration of fiber bedding bending is more likely to lead to interlayer cracking damage.

## 4. Conclusions

In view of the typical layered defects and wrinkle defects in the main spar structure of wind turbine blades, this paper uses the vacuum perfusion process (VARI) to fabricate GFRP composite materials with artificial prefabrication defects, and describes the manufacturing process and artificial prefabrication defect method of the main spar composite materials of wind turbine blades in detail. It provides an experimental reference for subsequent research on the damage evolution identification of wind power blade girder composites.

Acoustic emission experiments are used to analyze the mechanical properties and acoustic emission characteristics of wind turbine blade girder composites with various flaws under tensile loading. In addition to affecting the mechanical characteristics of composite materials, the presence of engineered defects will also impact the damage progression of material. For delamination defects and wrinkle defects, the different size of defects and the position of the layer will affect the damage evolution trend of the material.

The characteristic frequencies of the damage modes of GFRP composites were identified by K-means clustering analysis of acoustic emission parameters. The types of damage modes of wrinkle defects and delamination defects are the same, and the range of characteristic frequency is roughly the same. It is worth noting that the existence of defects will lead to certain fluctuations in the range of cluster center frequency and characteristic frequency, which should be paid attention to in the health monitoring of damage characteristics. The fracture failure mechanism of the material is mainly caused by a large number of fiber breakages and longitudinal splitting, rather than interface debonding.

## Figures and Tables

**Table 1:** Cluster analysis results of delamination defect specimens.

	Specimen A1		Specimen A2	
	Amplitude/dB	Center frequency/kHz	Amplitude/dB	Center frequency/kHz
Cluster1	40.1-91.8	71.31	40.1-91.5	63.20
Cluster2	40.3-86.5	131.84	40.3-83.2	142.87
Cluster3	41.5-72.2	297.60	41.3-71.5	254.46
Cluster4	42.2-66.3	407.25	41.6-67.8	365.20
	Specimen A3		Specimen A4	
	Amplitude/dB	Center frequency/kHz	Amplitude/dB	Center frequency/kHz
Cluster1	40.1-92.3	65.80	40.1-91.2	65.41
Cluster2	40.3-86.5	136.90	40.3-86.5	138.62
Cluster3	41.6-68.4	284.30	41.7-70.2	267.94
Cluster4	42.5-62.3	397.75	42.2-65.3	385.83

**Table 2:** Cluster analysis results of wrinkle defect specimens.

	Specimen Z1		Specimen Z2		Specimen Z3	
	Amplitude/dB	Center Frequency/kHz	Amplitude/dB	Center Frequency/kHz	Amplitude/dB	Center Frequency/kHz
Cluster1	40.1–89.2	71.65	40.1–91.2	73.22	40.1–92.3	69.38
Cluster2	40.5–90.3	144.42	40.3–86.5	148.53	40.3–86.5	137.24
Cluster3	41.0–75.8	284.25	41.7–70.2	281.61	41.6–68.4	287.20
Cluster4	42.2–57.3	398.65	42.2–65.3	392.83	42.5–62.3	402.27

**Table 3:** Acoustic emission events and energy percentages of different defective specimens.

Specimens	Percentage of Acoustic Emission Impacts (%)				Percentage of Acoustic Emission Energy (%)			
	40~55 dB	55~70 dB	70~85 dB	85~90 dB	40~55 dB	55~70 dB	70~85 dB	85~90 dB
A2	65.1	27.3	7.1	0.5	0.3	3.5	25.8	70.4
A3	61.9	29.3	8.1	0.7	0.8	3.8	26.6	68.8
A4	63.5	27.7	8.2	0.6	0.6	4.8	25.4	69.2
Z1	66.1	25.7	7.7	0.5	0.7	4.2	23.2	71.9
Z2	67.9	24.1	7.6	0.4	0.5	3.8	28.2	67.5
Z3	68.9	23.9	6.8	0.4	0.9	5.2	25.7	68.2

## Data Availability

The original contributions presented in the study are included in the article, further inquiries can be directed to the corresponding author.
